# Ada-WHIPS: explaining AdaBoost classification with applications in the health sciences

**DOI:** 10.1186/s12911-020-01201-2

**Published:** 2020-10-02

**Authors:** Julian Hatwell, Mohamed Medhat Gaber, R. Muhammad Atif Azad

**Affiliations:** Birmingham City University, Curzon Street, Birmingham, B5 5JU UK

**Keywords:** Explainable AI, Computer aided diagnostics, AdaBoost, Black box problem, Interpretability

## Abstract

**Background:**

Computer Aided Diagnostics (CAD) can support medical practitioners to make critical decisions about their patients’ disease conditions. Practitioners require access to the chain of reasoning behind CAD to build trust in the CAD advice and to supplement their own expertise. Yet, CAD systems might be based on black box machine learning models and high dimensional data sources such as electronic health records, magnetic resonance imaging scans, cardiotocograms, etc. These foundations make interpretation and explanation of the CAD advice very challenging. This challenge is recognised throughout the machine learning research community. eXplainable Artificial Intelligence (XAI) is emerging as one of the most important research areas of recent years because it addresses the interpretability and trust concerns of critical decision makers, including those in clinical and medical practice.

**Methods:**

In this work, we focus on AdaBoost, a black box model that has been widely adopted in the CAD literature. We address the challenge – to explain AdaBoost classification – with a novel algorithm that extracts simple, logical rules from AdaBoost models. Our algorithm, *Adaptive-Weighted High Importance Path Snippets* (Ada-WHIPS), makes use of AdaBoost’s adaptive classifier weights. Using a novel formulation, Ada-WHIPS uniquely redistributes the weights among individual decision nodes of the internal decision trees of the AdaBoost model. Then, a simple heuristic search of the weighted nodes finds a single rule that dominated the model’s decision. We compare the explanations generated by our novel approach with the state of the art in an experimental study. We evaluate the derived explanations with simple statistical tests of well-known quality measures, precision and coverage, and a novel measure *stability* that is better suited to the XAI setting.

**Results:**

Experiments on 9 CAD-related data sets showed that Ada-WHIPS explanations consistently generalise better (mean coverage 15%-68%) than the state of the art while remaining competitive for specificity (mean precision 80%-99%). A very small trade-off in specificity is shown to guard against over-fitting which is a known problem in the state of the art methods.

**Conclusions:**

The experimental results demonstrate the benefits of using our novel algorithm for explaining CAD AdaBoost classifiers widely found in the literature. Our tightly coupled, AdaBoost-specific approach outperforms model-agnostic explanation methods and should be considered by practitioners looking for an XAI solution for this class of models.

## Background

### Introduction

Medical diagnosis is a complex, knowledge intensive process. A medical expert must consider the symptoms of a patient, along with their medical and family history including complications and co-morbidities [1]. The expert may carry out physical examinations and order laboratory tests and combine the results with their prior knowledge. These activities are time intensive and, increasingly, considered sources of Big Data [2, 3]. Suitably experienced, available practitioners and experts are needed to orchestrate and interpret the results, yet these experts are a scarce resource in many healthcare settings. As healthcare needs grow and the sources of medical data increase in size and complexity, the diagnostic process must scale to meet these growing demands.

State of the art machine learning (ML) methods underpin many computer aided diagnostics (CAD) systems. CAD can address the aforementioned scalability challenges and may improve patient outcomes [4–6]. These ML methods demonstrate exceptional predictive and classification accuracy and can handle high dimensional data sets that often have very high rates of missing values. Examples of such challenging data sets include high throughput bioinformatics, magnetic resonance imaging scans, microarray experiments, and complex electronic health records (EHR) [7, 8], as well as unstructured, user-generated content (e.g. from social media feeds) that have been used to learn individuals’ sub-health and mental health status outside of a clinical setting [9, 10]. Unfortunately, however, many state of the art ML models are so-called “black boxes” because they defy explanation. The complexity of black box models renders them opaque to human reasoning. Consequently, experts and medical practitioners are reluctant to accept black box models in practice since they need to reason about, verify and approve the model’s output before making a final decision. In the clinical setting, the model’s output should facilitate professional decision-making alongside their expert clinical training and experience. A standalone classification from a black box model does not serve this purpose well, if at all. This barrier to adoption is evident, even when the black box models are demonstrably more accurate [1, 11–17]. There is also a legal right to explanation for high stakes decisions, which includes medical diagnosis and treatment recommendations [5, 18].

Some might argue that a black box model is no less transparent than a doctor [19]. Nevertheless, a doctor can be asked to justify their diagnosis and will do so from a position of domain understanding. In contrast, providing explanations for black box models is a very complex challenge. These models find patterns in data without domain understanding. Yet we wish to communicate explanations to a variety of levels of domain expertise: patient, practitioner, healthcare administrators and regulators. Additionally, we set higher standards of statistical rigour before granting our trust to ML derived decisions and explanations [20, 21].

Recent studies found that classification is the most widely implemented ML task in the medical sector and solutions using the AdaBoost algorithm [22] form a significant subset of the available research. Clinical applications include the diagnosis of Alzheimer’s disease, diabetes, hypertension and various cancers [23–26]. There are also non-clinical assessments of self-reported mental health, and subhealth status. The latter is characterised by chronic fatigue and infirmity that often leads to future ill-health. These non-clinical approaches used unstructured, user generated content from online health communities [9, 10]. AdaBoost has also been used as a preprocessing tool to select automatically the most important features from high dimensional data [27, 28]. Yet, AdaBoost is considered a typical black box as a consequence of its internal structure: an ensemble of typically 100s to 1000s of shallow decision trees. The ensemble uses a weighted majority vote to classify data instances; a system that is difficult to analyse mathematically. The widespread adoption of AdaBoost in medical applications, coupled with its black box nature leads to the challenge; to make AdaBoost explainable.

We present *Adaptive-Weighted High Importance Path Snippets* (Ada-WHIPS), a novel method for explaining multi-class AdaBoost classification through inspection of the model internals; a collection of adaptive weighted, shallow decision trees. The method proceeds by extracting the decision path from each tree that is specific to the data instance requiring an explanation (the explanandum). Only the paths that agree with the weighted majority vote are retained. These paths are disaggregated into individual decision nodes (which we call path snippets), and the weights are reassigned according to depth within the tree and frequency within the ensemble. The most important snippets are filtered and sorted by the newly applied weights. These adaptive-weighted, high importance path snippets are then greedily added to a classification rule. The final rule is tested for quality metrics and counterfactual conditions against the training (or historical) data.

To demonstrate our contribution, we now present four illustrative examples of Ada-WHIPS explanations. These examples have been drawn at random from the data sets used in our experiments, which are all CAD or medically relevant ML problems. An Ada-WHIPS explanation is a simple, conjunctive classification rule, presented alongside confidence and counterfactual (contrast) information. This includes: generality (coverage), specificity (precision), and how much precision decreases (% points) when any single rule term is violated. The end user can immediately determine the essential attributes (the features and decision boundary) that led to the model’s confident classification:

In Table 1, statistical features computed from foetal cardiotocograms are used to diagnose heart abnormalities. In Table 2, an online health community (self-selecting) responded to a twenty-four question survey on their mental health. The classification model identifies those individuals who have actually sought treatment. The individual shown in the examples has responded that they are experiencing problems at work and that there may be a family history of mental illness. Table 3 shows attributes from an EHR that were critical in determining the risk of readmission for one particular patient. Table 4 shows the results of a classifier for abnormal thyroid conditions. Full details of the data sets used can be found in Table 6.

**Table 1:** Explanation of a classifier for foetal heart abnormalities

Decision:	Explanation:	Contrast:	Confidence:
Normal	DP ≤0.0013 ∧	−74.5%	Coverage: 60.0%
	ALTV ≤7.7 ∧	−43.2*%*	Precision: 98.2% of covered
Prior 79.0%	Min ≤113.15	−34.58%	

DP: Number of prolonged decelerations per second.

ALTV: % time with abnormal short term variability.

Min: Minimum of baseline foetal heart rate histogram

**Table 2:** Explanation of a non-clinical mental health assessment classifier

Decision:	Explanation:	Contrast:	Confidence:
Has sought	work interfere ≤1.5 ∧	−45.6%	Coverage: 24.9%
treatment	family history >0.9	−23.3%	Precision: 94.6% of covered
Prior 54.9%			

Work interfere: If you have a mental health condition, do you feel it interferes with your work?

Answers: 0 = Often, 1 = Sometimes, 2 = Not Sure, 3 = Rarely, 4 = Never

family history: Do you have a family history of mental illness?

Answers: 0 = No, 1 = Not Sure, 2 = Yes

**Table 3:** Explanation of automated 30-day hospital readmission risk assessment

Decision:	Explanation:	Contrast:	Confidence:
Risk: Low	# inpatient ≤1.0 ∧	−58.1%	Coverage: 16.5%
	# emergency ≤0.5 ∧	−46.7%	Precision: 98.1% of covered
	# outpatient ≤0.5 ∧	−41.8%	
Prior 65.0%	# diagnoses ≤5.5	−39.6%	

# xxxx: number of e.g. hospital visits of type xxxx

**Table 4:** Explanation of a classifier for thyroid condition

Decision:	Explanation:	Contrast:	Confidence:
Abnormal	TSH >6.83	−78.5%	Coverage: 8.2%
			Precision: 98.2% of covered
Prior 26.0%			

TSH: Thyroid Stimulating Hormone level test result

We proceed with a walk through of the interpretation of Table 1: The model has classified the instance as "Normal." This is on a prior of 79.0% Normal in the training (historical) data. However, the given instance has a set of readings that raises the precision to 98.2%. If an almost identical instance were found with a point change in any one of the features listed (taking the instance outside the decision boundary), precision would decrease by the amount shown on the adjacent Contrast column. The new values would be worse than a random guess on this prior, with a raised number of prolonged decelerations per second returning a different outcome code altogether. These conditions hold on 60% of the historical data, making this a high quality rule that can inform the clinician’s decision on whether any intervention is necessary – most likely not, in this case.

The rest of this paper is organised as follows: We continue this Background section with an in-depth review of the current state of the art in XAI, related work in CAD and a recap of the Multi-Class AdaBoost algorithm. We introduce our novel algorithm and describe our experimental setup in the Method section. We report our results and elaborate on their significance in the Results section. Further important points are presented in the Discussion section. The article finishes with a section on Conclusion & future work.

### XAI and interpretable models - current state of the art

Medical practitioners making safety critical decisions need explanations of ML classification results that provide the required level of accountability. The current research seeks to address the challenge posed by the use of AdaBoost models in healthcare applications. In contrast to model-agnostic methods that operate on input sensitivity to synthetic data, our approach is to “open the black box” of an already trained and well performing AdaBoost model. This approach provides explanations that directly relate to the model internals. In the following paragraphs, we outline the state of the art and the novelty of our approach.

The decompositional approach [29] to interpretability is well established. “Decompositional” refers to the process of querying directly the smallest information unit of a model, e.g. the set of all decision nodes within each decision tree of an ensemble. Examples in the literature include: DefragTrees [30], Forex++ [31], RF+HC [32], inTrees [33], RuleFit [34], Brute [35]. All these methods generate a cascading rule list (CRL) as a simpler, surrogate of the original classification model. The prevalence of CRL as interpretable models indicates the importance of logical rules for explainability. Logical rules are intuitive to understand, being the standard language of reasoning [20, 36] and are the paradigm that we have adopted in our method.

The above mentioned methods are examples of globally interpretable proxy models; they allow the user to infer some understanding of the black box model’s overall behaviour. However, with such proxy models there is always a trade-off; increasing interpretability but also increasing classification error and giving no guarantees of fidelity with the original model. Anything less than perfect fidelity means that, for some instances, proxy and model do not agree. Explanations that refer to a different class than the model’s predicted class are of no use in a safety-critical setting, such as CAD. Ada-WHIPS uses logical rules and is a decompositional method but unlike the above mentioned methods, Ada-WHIPS explains one classification instance at a time rather than the global model behaviour described. The method is local and post-hoc [37]. Ada-WHIPS also has perfect fidelity by design. That is, the explanation generating process begins with the black model’s classification as its starting point and is, therefore, guaranteed to match.

Several post-hoc, per instance explanation methods have been proposed as model-agnostic frameworks (also known as didactic methods [29]). The model-agnostic assumption is that any model’s behaviour can be explained given unfettered access only to the model inputs and outputs (that is, to make an unlimited number of calls) but no access to the training data nor the model internals. Model-agnostic methods probe the model’s behaviour by generating a large, synthetic input sample. Each explanation is inferred from the effect of different input attributes on the outputs. Local Interpretable Model-agnostic Explanations (LIME) [21] generates a sparse linear model, SHapley Additive exPlanations (SHAP) [38] uses a game theoretic approach for a similar result: a set of non-zero coefficients for the input attributes. The coefficients are additive and their magnitude is proportional to the importance in the classification of the attributes they represent. As a result, these methods are categorised as Additive Feature Attribution Methods (AFAM) [38]. The main disadvantage of AFAM is that it is difficult to know when to apply an AFAM explanation to another previously unseen instance that does not share all of the same attribute values associated with the coefficients. Anchors [36] and LOcal Rule-based Explanations (LORE) [39] also use synthetic samples but generate a single classification rule (CR) as an explanation (as opposed to the many rules in a CRL). A CR-based explanation resolves the main disadvantage of AFAM because it is trivial to generalise a CR to another instance; the rule either covers or does not. Anchors uses the same synthetic sampling technique used by LIME since it was developed by the same research team to overcome the shortcoming of AFAM. LORE uses a genetic algorithm to generate the synthetic sample but this requires a very large number of calls to the black box model, and is computationally expensive to run in its own right.

Model-agnostic techniques, while effective in image and text classification, have disadvantages on tabular data sets. For one thing, they require additional checks; variance in the sampling process can cause variance in the resulting explanations over repeated trials [40, 41]. Furthermore, for tabular data, a realistic synthetic distribution must be estimated from the training data set or a large i.i.d. sample. This requirement violates the model-agnostic assumption of accessing only the inputs and outputs of the black box model. LIME, Anchors, and SHAP sample from the marginal training distribution, while LORE explores the marginal input domains. Clearly such synthetic samples have no guarantees to represent the underlying population because they do not use the joint distribution. In most real-world problems, the joint distribution is unknown or intractable. Yet, these methods explicitly access the training data but there is no rationale given in the relevant articles for not using the empirical distribution, for example by the bootstrapping method used in Brute [35]. Consequently, these model-agnostic methods are thought to put too much weight on unlikely or impossible examples. Moreover, LIME and Anchors require all features of tabular data to be categorical. Continuous features must be discretised in advance of training the classification model. To this end, quartile binning [36] is proposed by the authors. This is an arbitrary procedure and a significant compromise that puts constraints on the model of choice and potentially loses important information from the continuous features.

Ada-WHIPS, in contrast, assumes access to both the model internals and the training data. By decomposing the internals, using the adaptive weights and executing a greedy heuristic against the bootstrapped training data, the output explanation is an open-the-box method, and uses the empirical distribution instead of a synthetic distribution. Furthermore, Ada-WHIPS exploits the information-theoretic discretisation of the continuous features that occurs when the individual decision trees are induced during the AdaBoost model training. This information preserving approach is an advantage over the methods that require discretisation as a preprocessing step. Model-agnostic methods can also be slow to compute. For example, computing Shapley Values entails solving a large combinatorial problem which limits the scalability [42], while LORE’s synthetic samples are generated by a genetic algorithm that is not parallelisable in the currently available version^1^. Ada-WHIPS is fast, as our experimental study shows.

We suggest that the model-agnostic assumption should be taken with caution. There is a prevailing view in the XAI research community that model-agnostic methods are a very active research area while model-specific methods may be in decline. Yet, in a recent, comprehensive literature review [43] the following methods were categorised as model-agnostic when, in fact, they are model-specific: Saliency Maps, Activation Maximisation, Layer-wise Relevance Propagation. These methods all require access to the internal neurons in an Artificial Neural Network and their categorisation as model-agnostic may be a sign of confirmation bias in the research community. We also argue that model-agnostic methods are only required for a subset of ML problems, such as model auditing by an external third party. This scenario does not apply in CAD system development where the capability to add explanations would come from the owners themselves of the model and data. With access to both the training data and the model, decompositional methods should always be considered since they do not rely on synthetic data and can deliver explanations that are more representative of the model’s internals [43]. Treeinterpreter [44] is possibly the earliest model-specific explanation method, applicable to regression problems with Random Forest models. TreeSHAP [42], based on the SHAP method, assumes an underlying XGBoost model and queries the internal decision nodes. This model-specific design provides faster and more consistent results than the original SHAP algorithm for XGBoost models. Thus, model-specific methods are and should remain an active and relevant research area.

Finally, very few XAI methods have so far implemented counterfactuals, which are “what if” scenarios that indicate minimal changes to the inputs that would yield a different classification. LORE is the only well-cited example to the best of our knowledge and applies a strict change-of-class counterfactual paradigm and only works for binary classification. Ada-WHIPS provides a more flexible counterfactual solution that shows how the confidence (specificity) of a classification changes, as opposed to a discrete change of class. This novel, probabilistic approach allows the expert user to control and interpret the results since a decreasing confidence has ramifications even if the outcome code does not change. For example CAD may involve rare conditions in very unbalanced data sets, thus simply decreasing the probability that the individual is disease free may be enough to suggest an intervention. The method works just as well for multi-class problems.

As a minor contribution, we also provide a novel method to avoid over-fitting explanations that could potentially be applied elsewhere.

### Related work

CAD is an active research area. Yet, the safety critical nature suggests that it is unethical to make diagnoses without human intervention [45, 46]. XAI in healthcare offers the paradigm to assist rather than replace the medical expert. Hence, we present recent research that aligns to this paradigm. We focus on methods that predict or classify from non-image based clinical data. Table 5 summarises our review.

**Table 5:** Summary of related work

Author(s)	Date	Medical	Model	XAI
		Condition(s)		Mechanism
Lamy et al. [47]	2019	Breast Cancer	WkNN and MDS	CBR
		(treatment)		
Kwon et al. [48]	2018	General health	RNN	t-SNE and
				Visual Analytics
Adnan and Islam [31]	2017	Heart disease,	Tree ensembles	Logical Rules
		dementia		
Jalali and Pfeifer [8]	2016	Cancer	L1-SVM	Feature
		biomarkers	ensemble	importance
Turgeman and May [12]	2016	Hospital	C5.0 Tree and SVM	Logical Rule
		readmission	ensemble	
Jovanovic et al. [11]	2016	Hospital	Tree Lasso	Regression
		readmission		Coefficients
Letham et al. [13]	2015	Stroke	BRL	Bayesian Rules
Caruana et al. [6]	2015	Pneumonia risk	GA^2^M	PI plots
Kästner et al. [49]	2012	Breast cancer	Neural Gas	Fuzzy Rules

**Table 6:** Data sets used in the experiments

Data set	Target	Classes	Class balance	Features	Of which nominal	N
Breast cancer	mb	2	0.63:0.37	31	1	569
Cardiotocography	NSP	3	0.78:0.14:	22	1	2126
			0.08			
Diabetic retinopathy	dr	2	0.53:0.47	20	1	1151
Cleveland heart	HDisease	2	0.54:0.46	14	8	303
Mental health survey ’16	mh2	2	0.50:0.50	46	44	1433
Mental health survey ’14	treatment	2	0.51:0.49	24	3	1259
Hospital readmission	readmitted	2	0.54:0.46	65	1	25000
Thyroid	diagnosis	2	0.74:0.26	30	3	9172
Understanding society^2^	mh	3	0.22:0.62:	330	246	11745
			0.16			

^2^This data set is safeguarded by the UK Data Service and used under end user license. It is not included in our repository

Lamy et al. [47] uses a case-based reasoning (CBR) approach to recommend treatments for breast cancer patients. Using a combination of weighted k-nearest neighbours (WkNN) and multidimensional scaling (MDS), the user is presented with a visual interface making recommendations based on similarities/differences with historical cases. CBR provides the medical expert with several comparison instances/cases to evaluate, while Ada-WHIPS presents one classification rule directly extracted from the model internals that must be true of the explanandum instance while coverage statistics measure the rule’s generalisation to other instances.

Kwon et al. [48] presents RetainVis, a visual analytics application for predicting health status from health insurance data. Feature attribution values and t-SNE clustering are used to provide an interactive interface. The paper demonstrates the benefits and deeper insights available from tight coupling to a specific model; a recurrent neural network (RNN), in this case.

Adnan and Islam [31] uses a novel algorithm to simplify an existing tree ensemble. The compact, surrogate model is a rule list that can be used for classifying unseen instances. The authors claim that the global behaviour of the compact model is easier to interpret than the black box ensemble but the rule list can itself be long and time consuming to interpret. In contrast, our method is concerned with generating a single rule to explain a single instance at a time.

Jalali and Pfeifer [8] use an ensemble of linear support vector machines (L1-SVM) to predict cancer diagnosis and identify important patterns of gene expression. This novel approach is tightly coupled to the data domain (genetic biomarkers) whereas Ada-WHIPS could feasibly be applied to any tabular data including those not related to medicine or healthcare.

Turgeman and May [12] propose a simple ensemble of a C5.0 decision tree and a support vector machine (SVM). The easiest to classify instances can be explained by traversing the tree, while hard to classify instances are left to the SVM which remains a black box. Consequently, this method cannot produce a straightforward explanation for all instances, unlike our method.

Jovanovic et al. [11] implement a Tree-Lasso system for introducing domain knowledge about serious disease conditions into a sparse logistic regression model that is easy to interpret. Lasso based methods discover a small set of important features using *L*_1_-norm regularisation but the tree-lasso requires domain knowledge to be provided apriori. Ada-WHIPS rule conditions are discovered by information theoretic tree induction during the AdaBoost model training, and does not require any apriori inputs.

Letham et al. [13] proposes a novel interpretable model, the Bayesian Rule List (BRL). The model is used in stroke prediction. The predictive results are competitive with state of the art, but in common with cascading rule lists, interpretability decreases with rule depth as all previous rules must be considered and excluded. Ada-WHIPS generates one rule for one instance from a pre-trained AdaBoost model.

Caruana et al. [6] uses generalised additive models (GAM) allowing second order interaction (GA^2^M) to predict pneumonia risk and hospital readmission. GAMs inherently provide partial independence (PI) plots, giving insight into the global model behaviour, and excellent predictive results. Domain knowledge was required apriori to discretise several features and to determine which second order interactions to include. However, interpretation of the non-linear components remains a challenge. Our method is a completely different approach that provides an explanation for individual cases and requires no apriori domain expertise.

Kästner et al. [49] integrates expert knowledge into a neural gas. Interpretability arises from the activation of the explicitly incorporated fuzzy rules. The outputs of this novel method includes scored rule conditions but the fuzzy rules must be introduced apriori, again in contrast to Ada-WHIPS that requires no apriori domain knowledge.

### Multi-Class adaBoost

In this section, we describe multi-class AdaBoost, with which our method is tightly coupled. Boosting is a method for generating a strong classifier by sequentially combining weak, base classifiers. It is one of the most significant developments in Machine Learning [50, 51]. AdaBoost [52] was the first, widely used implementation of boosting and is still favoured for its accuracy, ease of deployment and fast training time [53–55]. It uses shallow decision trees as the base classifiers. On each iteration, the training sample is re-weighted such that the next decision tree focuses on examples that were previously misclassified, while previously generated classifiers remain unchanged (the details of this iterative re-weighting are not central to this research so we refer the interested reader to [52, 56]). AdaBoost also adaptively updates its base classifier weights based on their individual performance, which we discuss now in further detail. Two algorithms, Stagewise Additive Modeling using a Multi-class Exponential loss function (SAMME) and real-valued SAMME (SAMME.R) [56] have emerged as the standard [57] for extending the original AdaBoost algorithm from binary classification to multi-class problems. The following formulations are based on [56].

Let $f : \mathcal {X} \longmapsto \mathcal {Y}$ be an unknown classification function that we would like to approximate, where $\mathcal {X}$ is an $\mathbb {R}^{d}$ input space and $\mathcal {Y} = \{C_{1},\ \dots,\ C_{K} \}$ is the set of possible classes. Let **X** be an input data set and our multi-class AdaBoost model be *g*(**X**)≈*f*(**X**). To classify an instance **x**, the output of a SAMME model is the weighted majority vote of all the base classifiers.

1 $$\begin{array}{*{20}l} g(\mathbf{x}) = C_{k},\ k &= {arg}\underset{k \in K}{{max}} \sum^{M}_{m=1} \alpha^{(m)} \cdot T^{(m)}(\mathbf{x}),\\ T^{(m)}(\mathbf{x}) &= [c_{1},\ \dots,\ c_{K}],\ \sum T^{(m)}(\mathbf{x}) = 1 \end{array} $$

where $[c_{1},\ \dots,\ c_{K}]$ is a one dimensional (1D) vector indicating the position of the output class and is the output of a single tree *T*^(*m*)^ at iteration *m*. Within this 1D vector, *c*_*k*_=1, *c*_*j*_=0, *j*≠*k* indicates that *C*_*k*_ is the predicted class. The whole model $g = \left \{\left \{T^{(1)},\ \dots,\ T^{(M)}\right \},\ \left \{ \alpha ^{(1)},\ \dots,\ \alpha ^{(M)} \right \} \right \}$ is the combination of a set of *M* base decision tree classifiers and a set of *M* classifier weights. These weights are calculated during the training phase as:

2 $$ \alpha^{(m)} = \log \frac{1 - {err}^{(m)}}{{err}^{(m)}} + \log(K - 1),\ 0 < {err}^{(m)} \leq 1 - \frac{1}{K}  $$

where *err*^(*m*)^ is the error rate at iteration *m*.

To classify an instance **x** with SAMME.R, each base classifier returns a vector of the conditional probabilities that the class of **x** is *C*_*k*_. This is the distribution of training instance weights in the terminal node of the decision path taken by **x** through each tree:

3 $$ {\begin{aligned} T^{(m)}(\mathbf{x}) = [ \mathbb{P}_{T^{(m)}}(C_{1}|x),\ \dots,\ \mathbb{P}_{T^{(m)}}(C_{K}|x) ],\\ \sum T^{(m)}(\mathbf{x}) = 1,\ y \in \mathcal{Y} \end{aligned}}  $$

and confidence weights are calculated at run time as:

4 $$ {}\alpha^{(m)}_{k}|x = (K-1)\big(\log \mathbb{P}_{T^{(m)}}(C_{k}|x) - \frac{1}{K} \sum^{K}_{j=1} \log \mathbb{P}_{T^{(m)}}(C_{j}|x)\big).  $$

The output of the whole model is the majority vote based on the additive contribution of these confidence weights per class:

5 $$ g(\mathbf{x}) = C_{k},\ k = {arg}\underset{k}{{max}} \sum^{M}_{m=1} \alpha^{(m)}_{k}|x.  $$

where $g = \left \{T^{(1)},\ \dots,\ T^{(M)}\right \}$ (weights $\alpha ^{(m)}_{k}$ evaluated at run time).

## Method

### Ada-WHIPS

**Fig. 1:**
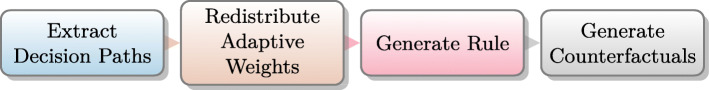
Conceptual diagram of Ada-WHIPS

We now present Ada-WHIPS, our algorithm for generating a CR based explanation for the classification of an explanandum instance **x** by a previously trained AdaBoost model *g*. The algorithm begins by initialising a rule as an empty antecedent and the classification outcome *g*(**x**) as the consequent. Thus, the CR always agrees with the black box, by design. The algorithm then proceeds through the steps shown in Fig. 1, to identify a small set of antecedent terms, or logical conditions. These conditions must be true of **x** and must exert the most influence on the classification result. The source of these logical conditions is the ensemble of decision trees that make up *g*. The influence is determined by the classifier weights within the internals of *g*, which themselves are derived from the error rates (weights increase as errors decrease).

#### Extract decision paths

**Fig. 2:**
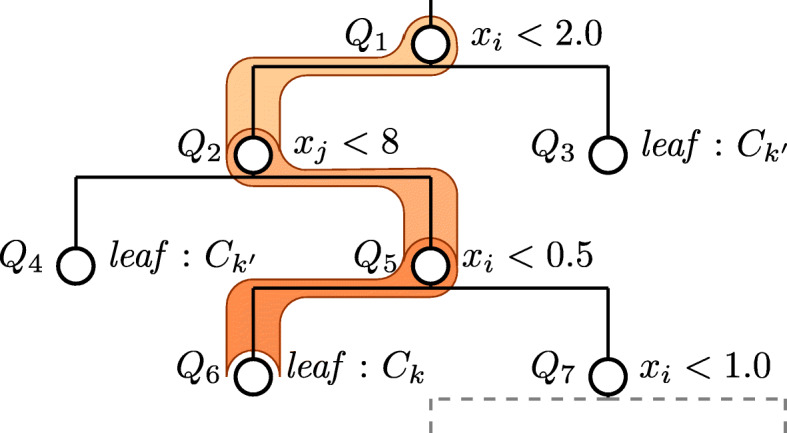
Conceptual diagram of a decision path for one instance through one tree

An AdaBoost model typically comprises 100’s-1000’s of shallow decision trees, potentially resulting in a very large search space. For a given **x**∈**X**, we can reduce this space logarithmically by considering only decision paths of that **x** in each decision tree and ignoring all other branches. The paths retain all the information about how *g*(**x**) was determined. A conceptual example of extracting the decision path is shown in Fig. 2. Here, $\mathbf {x} = \{\dots,\ x_{i} = 0.1,\ x_{j} = 10,\ \dots \}$, where *x*_*i*_ is the attribute value of the *i*^*th*^ feature. The decision path starts from the root node *Q*_1_, following the binary split conditions down to a leaf node. The decision path contains node detail triples of the following form (*j*,*ν*,*τ*), where *j* is a feature index and $\nu \in \mathbb {R}$ is the threshold for the inequality *x*_*j*_<*ν* and *τ*∈{0,1} is the binary truth of evaluating the inequality. Note that for this instance, all other nodes are irrelevant. For example, even though *Q*_7_ applies (*x*_*i*_<1.0), it cannot be reached by **x** because of the evaluation at *Q*_5_.

The search space can be further reduced by considering only those trees that agreed with the weighted majority vote. The rationale for this is based on the application of maximum margin theory to boosting [58]. If **x** is an unseen instance, the margin in SAMME is:

6 $$ \begin{aligned} {margin} &= \frac{a^{+} - a^{-}}{\sum^{T}_{m=1} \alpha^{(m)}},\ a^{+} = \sum^{|\mathcal{T}^{+}|}_{n=1} \alpha^{(n)},\ a^{-} = \frac{1}{K-1} \sum^{K}_{k = 1} \sum^{|\mathcal{T}^{-}|}_{u=1} \alpha^{(u)}, \\ \mathcal{T}^{+} &= \left\{T : g(\mathbf{x})= C_{k},\ k = {arg}\underset{k \in K}{{max}} T(\mathbf{x}) \right\},\\ \mathcal{T}^{-} &= \left\{T : g(\mathbf{x}) = C_{k},\ k \neq {arg}\underset{j \in K}{{max}} T(\mathbf{x}) \right\},\ T^{(m)}, \alpha^{(m)} \in g. \end{aligned}  $$

The quantity *a*^+^, represents the sum of weights from the classifiers that voted for the majority class and *a*^+^>*a*^−^ is always true for the majority class. The set $\mathcal {T}^{+}$ are the base classifiers that voted in the majority and thus contributed their weight to *a*^+^, and $\mathcal {T}^{-}$ are the remaining classifiers. $\mathcal {T}^{+}$ completely determines the ensemble’s output for a given instance because an ensemble classifier formed from the union of $\mathcal {T}^{+}$ and any subset of $\mathcal {T}^{-}$ would return the same classification with a larger *margin* because $a^{-}_{*} < a^{-},\ \mathcal {T}^{-}_{*} \subset \mathcal {T}^{-}$. We found no margin formalisation for SAMME.R in the literature but we can define $\mathcal {T}^{+} := \left \{ (T^{(m)}, \alpha ^{(m)}_{k}) : \alpha ^{(m)}_{k} \geq \alpha ^{(m)}_{j},\ k,j \in \{1,\ \dots,\ K\} \right \}$ and, as a convenience, we can substitute the *α* terms in Eq. (6) for the following Kullback-Leibler (KL) Divergence. The KL-Divergence (also known as “relative entropy”) measures the information lost if a distribution *P*^′^ is used, instead of another distribution *P* to encode a random variable and is defined as:

7 $$ D_{KL}(P \parallel P') = - \sum_{x \in \mathcal{X}} P(\mathbf{x}) \log \left(\frac{P(\mathbf{x})}{P'(\mathbf{x})} \right)  $$

and we set *P*,*P*^′^ as the posterior class distribution of each *T*^(*m*)^(**x**) given in Eq. (3), and prior class distribution in the training data, respectively. The KL-Divergence will be larger for trees that classify with greater accuracy, relative to the prior distribution. The *D*_*KL*_ emulates the classifier weights yielded by Eq. (2), which allows the rest of the algorithm to proceed in an identical manner for SAMME and SAMME.R.

#### Redistribute adaptive weights

To avoid a combinatorial search of all the available decision nodes, we sort them, prior to rule merging, according to their ability to separate the classes. To do this, we disaggregate the entire set of decision paths into individual decision nodes and redistribute the classifier weights onto the nodes. This procedure is illustrated in Algorithm 1. The contribution of each node is conditional on the previous nodes in the path and this sorting must take into account the node order in the originating tree. To do this, we apply Eq. (7) to determine the relative entropies at each point in a path. For each root node, we set *P*,*P*^′^ as the class distribution when applying that decision to the training data, and the prior class distribution respectively. For subsequent nodes, *P* is the class distribution after applying all previous decision nodes including the current node and *P*^′^ is the distribution up to but not including the current node. The relative entropy scores for nodes in a single path are normalised such that their total is equal to that of the classifier weight *α*^(*m*)^. The scores are grouped and summed for nodes that appear in multiple paths. We filter the nodes, keeping only those with the largest weights (e.g. top 20%). Finally, all nodes from all paths are sorted by this score in descending order.


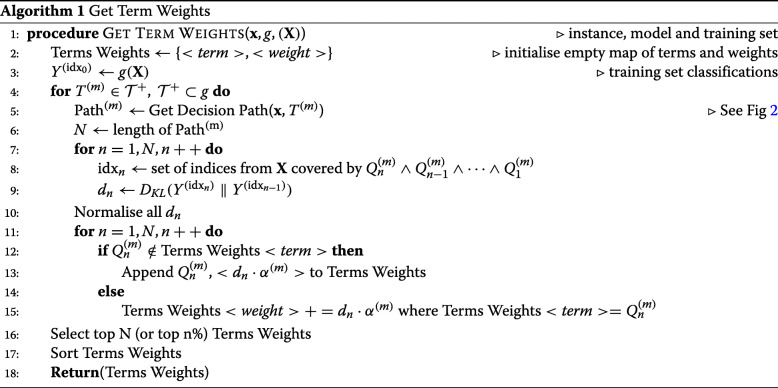


#### Generate classification rule

It is trivial to convert the node detail triples (*j*,*ν*,*τ*) into antecedent terms of a CR [59]. We use nodes and terms interchangeably from here on. The objective is to find a minimal set of terms that maximises both precision and coverage while mitigating the problem of over-fitting. Over-fitting can occur if we maximise precision as an objective function. We risk converging on “tautological” rules that provide no generalisation. This is because precision is trivially maximised by single instances. A tautological rule contains enough terms to identify a single instance uniquely. In a noisy data set, there could be many such local maxima. Therefore, we propose *stability* as a novel objective function, defined as:

8 $$ \zeta(\mathbf{x}, g, \mathbf{Z}) = \frac{|\{ \mathbf{z} : g(\mathbf{z}) = g(\mathbf{x}),\ \mathbf{z} \in \mathbf{Z} \}|}{|\mathbf{Z}| + K}  $$

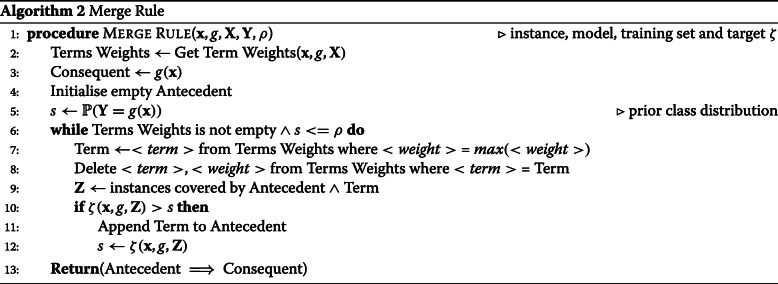


where **Z** is the set of instances covered by the current rule and *K* the number of classes. The maximum achievable *ζ* is $\frac {1}{K}$ for a single instance but approaches precision asymptotically as |**Z**|→*∞*. Stability, therefore acts as a brake on adding too many terms and over-fitting. We proceed with a breadth first search, iteratively adding terms to an initially empty rule. We always add the first term in the sorted list. Then, we work down the list, greedily adding further terms if they increase stability and discard them if they do not. The algorithm stops when a threshold stability (e.g. 0.95) is reached or the list is exhausted. These steps are illustrated in Algorithm 2.

#### Generate counterfactuals

**Fig. 3:**
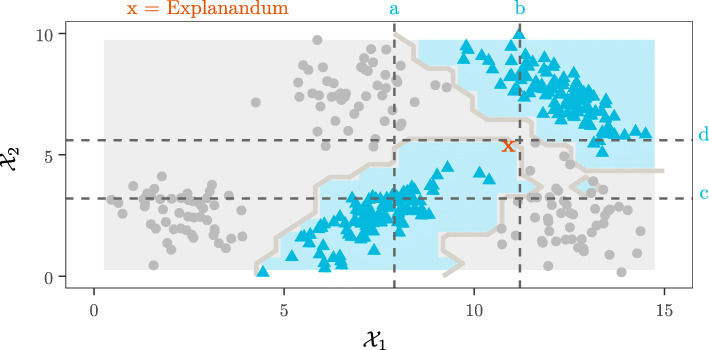
Counterfactual spaces - conceptual diagram

Counterfactuals answer the question “what would have happened if... ?” They illustrate minimal changes in the inputs that would give different results. Some authors define counterfactual (sometimes called contrastive) explanations as a minimal change set on the inputs that would return a different result [5, 15, 39, 60]. However, discrete change-of-classification counterfactuals do not allow any uncertainty. We suggest a fuzzy definition is better suited here; namely, if precision (specificity) decreases beyond a user-defined tolerance. The expert can better exercise their judgement with this approach. For example, decreasing from high to low confidence in a CAD or risk score can lead to requests for additional tests, a less aggressive clinical intervention and so on. Since the definition of counterfactuals is a minimal change set, it is not necessary (nor even practical) to provide every possible input scenario. It suffices to show the effect of each point change and this is easy to do with CR simply by changing each of the rule terms, one at a time. Any point changes that do not decrease the precision beyond the user-defined tolerance represent a non-counterfactual change and can be removed from the rule. This procedure provides an intuitive pruning mechanism for removing redundant terms that might have been added during the greedy rule merge algorithm. We illustrate this concept visually in Fig. 3. Here a model with a complex decision boundary is trained on a synthetic data set (a Gaussian mixture model) which has two classes, shown as triangles and circles. The model classifies an explanandum instance **x** as a triangle. The explanation is found - the following CR: $\{\mathbf {z} : a \leq z_{1} \leq b,\ c \leq z_{2} \leq d,\ \mathbf {z} \in \mathcal {X}\} \implies \text {triangle}$. The counterfactual spaces are those spaces immediately adjacent to the four rule boundaries, derived by reversing one inequality at a time:

9 $$ \begin{aligned} \big\{ &\{\mathbf{z} : z_{1} \leq a, c \leq z_{2} \leq d\},\ \{\mathbf{z} : b \leq z_{1}, c \leq z_{2} \leq d\},\\ &\{\mathbf{z} : a \leq z_{1} \leq b, z_{2} \leq c\},\ \{\mathbf{z} : a \leq z_{1} \leq b, d \leq z_{2}\},\ \mathbf{z} \in \mathcal{X} \big\} \end{aligned}  $$

Even though the triangle class is still predicted for parts of these spaces, the expected precision decreases drastically for a CR that is formed from any one of these counterfactual spaces for the antecedent and the same consequent. Thus, the original rule provides a crisp boundary where the maximal precision holds. The counterfactual rules communicate how much precision decreases when the rule is violated in any one dimension.

### Experimental design

We compared Ada-WHIPS in an experimental study with the state of the art. Three metrics are used to measure effectiveness, namely, coverage, precision and our new measure of stability. Efficiency, in terms of computing performance, is measured using the average time to generate an explanation. Comparisons are made against two other CR-based, per instance explanation methods: Anchors [36] and LORE [39]. Both methods are model-agnostic. Readers who are familiar with XAI research may question the omission of LIME [21] and SHAP [38], which are the most discussed per instance explanation methods. LIME and SHAP fall into a different class of methods, described as *additive feature attribution methods* (AFAM). AFAM are, effectively, local linear models (LM) whose coefficients relate the importance of various attributes to the original model’s classification of the explanandum. There is no obvious way to apply the local LM for one instance to any other instances in order to calculate the quality measures such as precision and coverage, and comparison with CR-based methods is of limited value [36]. Fortunately, Anchors has been developed by the same research group that contributed LIME and uses the same synthetic sampling technique. Anchors can be viewed as a rule-based extension of LIME and its inclusion into this experimental study provides a useful comparison to best in class AFAM research.

### Hardware setup

The experiments were conducted using Python 3.6.x running on a standalone Lenovo ThinkCentre with Intel i7-7600 CPU @ 3.4GHz and 32GB RAM using the Windows 10 operating system.

### Data sets

We used nine data sets described in Table 6. These were sourced from the UCI Machine Learning repository [61] and represent specific disease diagnoses from clinical test results, except; the mental health surveys (Kaggle) which represents case studies in detecting mental health conditions from non-clinical online health community data; the hospital readmission data (Kaggle) which represents a large EHR; and understanding society [62] which is from the General Population Sample of the UK Household Longitudinal Study and used under license. We use the file from waves 2 and 3 where participants had a health visit carried out by a qualified nurse. At least one study [63] has shown that the biomarkers measured in the survey may be associated with the results from self-completion instruments measuring mental health. We run a classification task for the SF-12 Mental Component Summary (PCS) which has been discretised into nominal values "poor," “neutral” and “good.’

### Limitations of the study

Unfortunately, we discovered that LORE was not scalable after finalising our experimental design. The time cost of generating a synthetic distribution by means of a genetic algorithm rendered the method unusable on some of the data sets. The time per instance was on average twenty-five to thirty minutes for the hospital readmission data set and more than two hours per instance on the understanding society data set. The method generated system errors on the mental health survey ’14 data set and was not runnable at all. We thoroughly examined the source code to look for opportunities to parallelise the operation, but the presence of a dynamically generated, non-serialisable distance function rendered this impossible. We have included the results where the method did run to completion.

### AdaBoost model training and testing

Each data set was split into training and test sets (70%, 30%) by random sampling without stratification or other class imbalance correction. We trained AdaBoost models using ten-fold cross-validation of the training set on number of trees ${ntrees} \in \{200,\ 400,\ \dots,\ 1600 \}$ and maximum tree depth parameter *maxdepth* was always 4. We used the *ntree* setting that delivered the highest classification accuracy to train a final model on the whole training set.

As mentioned in the section on related work, Anchors requires all features of the data to be categorical [36]. For our experiments, we generated a copy of each data set, and discretised them using Anchors’ provided quartile binning function. A second AdaBoost model was generated from this discretised data set for Anchors to explain. Training and test splits used identical indices as the undiscretised versions. Each test set was then used as the pool of unseen instances to be classified by the AdaBoost model and explained by Ada-WHIPS, Anchors and LORE. Thus, there are three comparable explanations for each test instance. Generating explanations is done instance by instance, not batch wise as in classification. So, for time constraints, the number of instances (test units) was limited to either the whole test set or the first one thousand test instances, whichever was the smaller. For each explanation, all the remaining instances from the entire test set were used to assess the standard quality measures, precision and coverage, along with the novel quality measure, stability (8), which is more sensitive to over-fitting. This leave-one-out procedure ensures that test scores are not biased by leakage of information from the explanation-generating instance. The entire procedure is repeated for SAMME and SAMME.R AdaBoost models.

We present the performance scores of the trained models in Table 7. It is important to note that the model training is part of the experimental setup and not to be taken as results per se. These training scores simply reflect the performance of AdaBoost; critiquing the performance of AdaBoost itself is not the objective of this work. We provide this level of detail only to demonstrate that the trained AdaBoost models reasonably approximate the underlying data sets and are very accurate. However, a true explanation by definition must stay faithful to the trained model regardless of whether the model is accurate or not (though a poor model would never be used in clinical practice). We show generalisation accuracy scores and Cohen’s *κ* for the two models (discretised and undiscretised data set variants). Cohen’s *κ* is a useful measure in multi-class problems and class imbalanced data because this statistic corrects for chance agreement, which can be high in such cases. Values close to zero indicate a high degree of chance agreement. See Appendix for further details on Cohen’s *κ*.

**Table 7:** Final AdaBoost model scores

		Undiscretised: used by		Discretised: used by	
		Ada-WHIPS & LORE		Anchors	
Data	ntree	Accuracy	*κ*	Accuracy	*κ*
SAMME					
Breast cancer	200	0.98	0.96	0.96	0.92
Cardiotocography	800	0.94	0.84	0.89	0.70
Diabetic retinopathy	1000	0.68	0.36	0.66	0.33
Cleveland heart	200	0.77	0.52	0.80	0.59
Mental health survey ’16	200	0.88	0.76	0.88	0.75
Mental health survey ’14	200	0.83	0.65	0.81	0.62
Hospital readmission	800	0.62	0.22	0.60	0.18
Thyroid	1200	0.97	0.92	0.80	0.45
Understanding society	600	0.64	0.13	0.61	0.14
SAMME.R					
Breast cancer	1000	0.98	0.96	0.95	0.90
Cardiotocography	1600	0.94	0.82	0.88	0.67
Diabetic retinopathy	200	0.69	0.38	0.65	0.30
Cleveland heart	400	0.76	0.50	0.82	0.63
Mental health survey ’16	800	0.87	0.73	0.86	0.72
Mental health survey ’14	200	0.80	0.60	0.81	0.63
Hospital readmission	200	0.62	0.22	0.63	0.23
Thyroid	1600	0.97	0.92	0.76	0.37
Understanding society	200	0.62	0.13	0.62	0.15

Accuracy and Cohen’s kappa on Held Out Data

### Significance testing

Our approach for the experimental study is based on the simulated user study implemented in [36]. In that study, coverage represents the fraction of previously unseen instances a user could attempt to classify after seeing an explanation and thence how generally the rule applies to the whole population. Similarly, precision represents the fraction of those classifications that would be correct if a user applied the explanation correctly, indicating the specificity of the rule. Real users who were shown high coverage and precision rule-based explanations demonstrated significantly improved task completion scores over those who were shown AFAM explanations.

To determine statistical significance, we report differences between precision, stability and coverage among the algorithms using non-parametric hypothesis tests. The reason for using these tests is that these measures are proportions; from the interval [0,1] and very right-skewed by design since each method tries to generate very high precision explanations. We use the paired samples Wilcoxon signed rank test where we have results for just Ada-WHIPS and Anchors. The null hypothesis of this test is that the medians of the two samples are equal and the alternative is that the medians are unequal. We use the Friedman test where we have results for all three methods. The Friedman test is a non-parametric equivalent to ANOVA and an extension of the rank sum test for multiple comparisons. The null hypothesis of this test is that there is no significant difference between the mean ranks of all the groups and the alternative is that at least two mean ranks are different. For all our three-way comparisons using the Friedman test, p-values were vanishingly small ≈0. So, in our report that follows, we proceed directly to the recommended pairwise, post-hoc comparison test with the Bonferroni correction (for three pairwise comparisons) proposed in [64]. It is sufficient for this study to demonstrate whether the top scoring algorithm was significantly greater than the second place algorithm on our quality measures of interest. The critical value for a two-tailed test with the bonferroni correction is $\frac {0.025}{3} = 0.00833$. See Appendix for further details on the Friedman test applied here.

The three-way post-hoc tests and the two-way comparisons are shown in separate tables to avoid drawing invalid comparisons. The mean rank, rather than the mean, is given in the tables, as this is the statistic compared between groups by the chosen tests. A significant result is indicated by ** and the winning algorithm is formatted in boldface only if the results are significant.

## Results

We begin by presenting the four worked examples from the introduction. Then, we assess the aggregated quality measures for the test samples. For each measure, we present dot chart showing the mean score (with standard errors) aggregated over all the test instances. In several cases, the results are close, resulting in over-plotting that could lead to confusion as to whether two or three results are returned for a given data set. To assist the reader in distinguishing the scores, a guide line has been added. However, each data set should still be viewed as a separate experiment.

### Worked examples

Tables 8, 9, 10 and 11 present the worked examples from our introduction. Readers are reminded that the paths taken by a single instance in a pre-trained AdaBoost model are disaggregated into individual decision nodes. The most important of these nodes are recombined into a high quality rule for explaining the model’s classification. Note that models had different numbers of iterations, and trees can grow to any depth up to the maximum of 4. It is also interesting to note a detail about the paths from trees that disagreed with the majority classification; that is, while they covered the instance (as they must), the boundary attributes are very distant from the instance attributes in the input space. We suggest that this is in keeping with the theoretical principles of AdaBoost – each iteration focuses on misclassified instances of the previous iteration, leading to a very different decision boundary in the next tree.

**Table 8:**
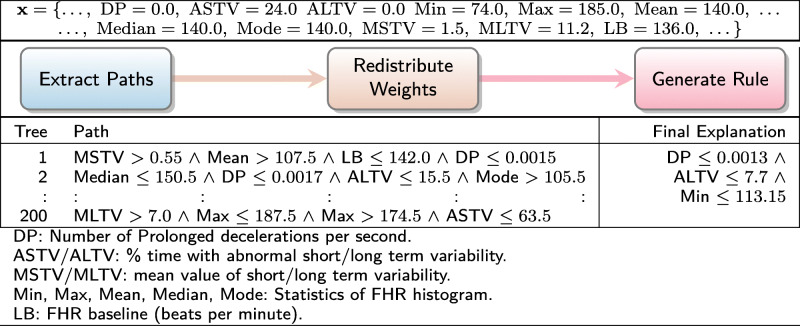
Worked example for foetal heart abnormalities data set

**Table 9:**
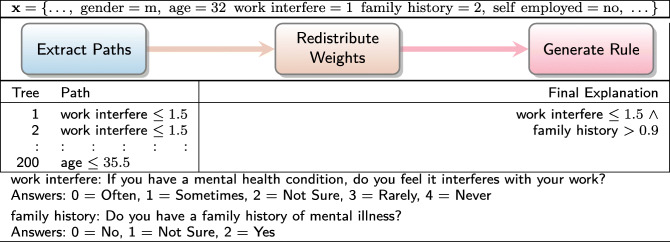
Worked example for non-clinical mental health assessment data set

**Table 10:**
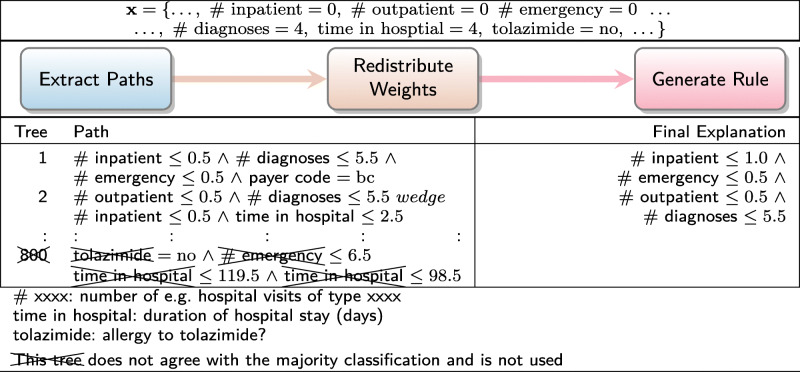
Worked example for automated 30-day hospital readmission risk assessment data set

**Table 11:**
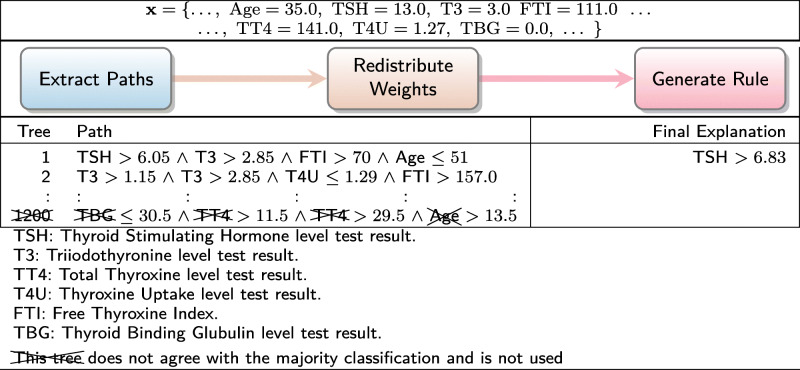
Worked example for thyroid condition data set

### Coverage analysis

**Fig. 4:**
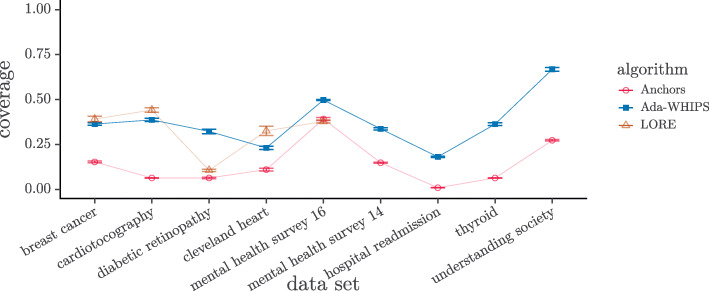
Mean Coverage for SAMME model explanations. Guide lines are added to mitigate over-plotting

**Fig. 5:**
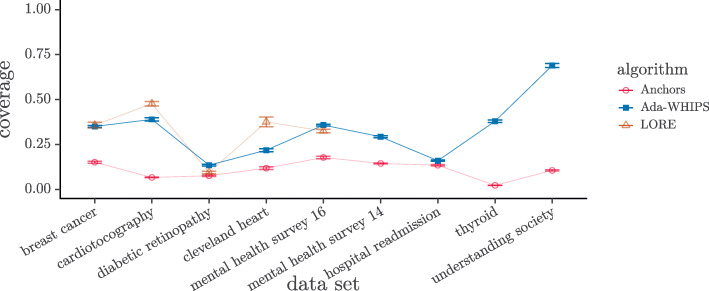
Mean Coverage for SAMME.R model explanations. Guide lines are added to mitigate over-plotting

We present a visual analysis of the raw data (see Appendix for results tables) and tabulate the results of our statistical tests. A cursory inspection of the mean coverage charts shown in Figs. 4-5 indicates that Anchors has the lowest mean coverage over all the data sets but the comparison between Ada-WHIPS and LORE is less clear cut. The results of the hypothesis tests are given in Tables 12-13. The Wilcoxon tests showed that Ada-WHIPS always has significantly higher coverage than Anchors. Ada-WHIPS was the top algorithm in all but three of the post-hoc tests for three-way comparisons and in the top two alongside LORE with no significant difference for the remaining tests.

**Table 12:** Coverage: Top two by mean rank (mrnk) for three-way comparisons

Data	1^st^	mrnk	2^nd^	mrnk	N	*z*	*p*.value
SAMME							
Breast	LORE	1.54	Ada-WHIPS	1.61	170	0.41	0.3412
Cardiotocography	LORE	1.52	Ada-WHIPS	1.62	637	1.06	0.1442
Diabetic retinography	**Ada-WHIPS**	1.39	LORE	2.20	344	6.76	≈0**
Cleveland heart	LORE	1.63	Ada-WHIPS	1.82	90	0.8158	0.2072
Mental health survey ’16	**Ada-WHIPS**	1.51	Anchors	2.22	429	6.19	≈0**
SAMME.R							
Breast	Ada-WHIPS	1.48	LORE	1.70	170	1.29	0.0980
Cardiotocography	LORE	1.52	Ada-WHIPS	1.62	637	1.14	0.1269
Diabetic retinography	**Ada-WHIPS**	1.57	Anchors	2.17	344	4.98	0.0000**
Cleveland heart	LORE	1.50	Ada-WHIPS	1.86	90	1.52	0.0649
Mental health survey ’16	Ada-WHIPS	1.68	Anchors	1.80	429	1.04	0.1492

**Table 13:** Coverage: Mean rank (mrnk) for two-way comparisons

Data	1^st^	mrnk	2^nd^	mrnk	N	*V*	*p*.value
SAMME							
Mental health survey ’14	**Ada-WHIPS**	1.16	Anchors	1.84	377	66	≈0**
Hospital readmission	**Ada-WHIPS**	1.01	Anchors	1.98	1000	782.5	≈0**
Thyroid	**Ada-WHIPS**	1.10	Anchors	1.90	1000	14806	≈0**
Understanding society	**Ada-WHIPS**	1.20	Anchors	1.80	1000	858	≈0**
SAMME.R							
Mental health survey ’14	**Ada-WHIPS**	1.13	Anchors	1.87	377	119	≈0**
Hospital readmission	**Ada-WHIPS**	1.33	Anchors	1.67	1000	174990	≈0**
Thyroid	**Ada-WHIPS**	1.02	Anchors	1.98	1000	1754	≈0**
Understanding society	**Ada-WHIPS**	1.07	Anchors	1.93	1000	6417	≈0**

### Precision analysis

**Fig. 6:**
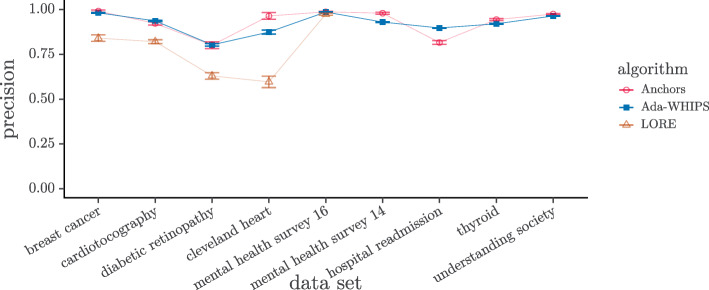
Mean Precision SAMME. Guide lines are added to mitigate over-plotting

**Fig. 7:**
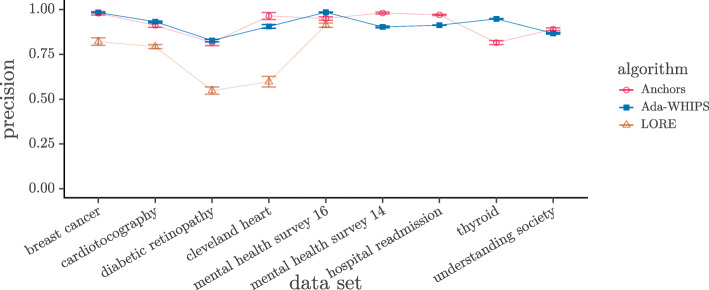
Mean Precision SAMME.R. Guide lines are added to mitigate over-plotting

The mean precision chart, (Figs. 6-7), show that LORE has the lowest precision in all but one of the data sets where LORE results are available. It is harder to see if there is a definitive lead between Ada-WHIPS and Anchors.

**Fig. 8:**
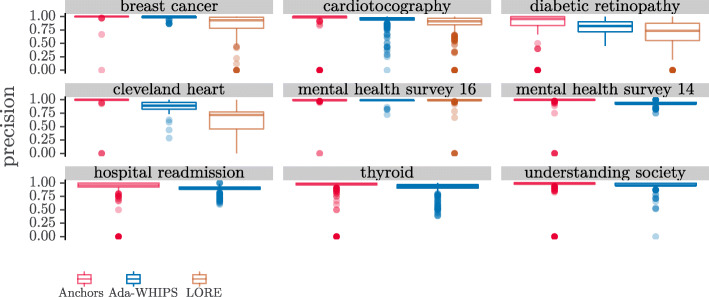
Distributions of Precision SAMME

**Fig. 9:**
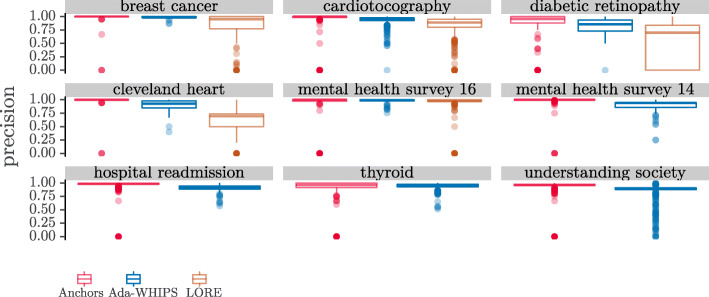
Distributions of Precision SAMME.R

However, the complete picture – and the cost to Anchors of implementing a precision guarantee – can be seen in the distribution charts in Figs. 8-9. Here we see that a certain proportion of explanations have a precision of 0.0. The result shows that Anchors (and LORE to a lesser extent) is over-fitting. Some explanations are so specific that they only explain the explanandum and do not generalise to other instances in the test set. We present the proportion of 0.0 precision explanations that were returned by each algorithm in Table 14.

**Table 14:** Proportion of over-fitting, 0.0 precision explanations

	SAMME			SAMME.R		
Data	Ada-WHIPS	Anchors	LORE	Ada-WHIPS	Anchors	LORE
Breast cancer	0	0.01	0.04	0	0.18	0.06
Cardiotocography	0.00	0.07	0.09	0.00	0.08	0.09
Diabetic retinopathy	0	0.15	0.19	0.00	0.13	0.28
Cleveland heart	0	0.03	0.14	0	0.03	0.12
Mental health survey ’16	0	0.00	0.01	0	0.04	0.06
Mental health survey ’14	0	0.01	N/A	0	0.01	N/A
Hospital readmission	0	0.15	N/A	0	0.01	N/A
Thyroid	0	0.03	N/A	0	0.15	N/A
Understanding society	0.00	0.01	N/A	0.01	0.08	N/A

**Fig. 10:**
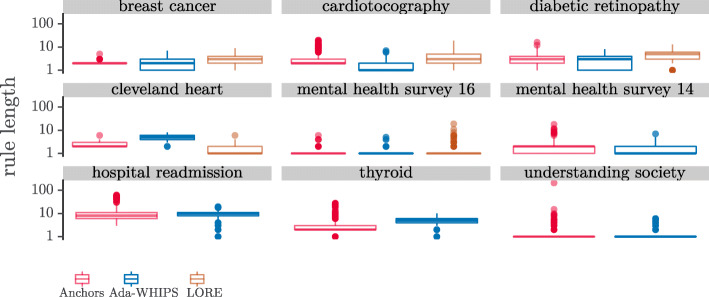
Distributions of Rule Length. Note the y-axis is log10 scaled

The proportions vary from around 0.5*%*−28*%*. There are important consequences for methods that suffer this level of over-fitting. The most important consequence is that 0.0 precision rules are so specific that they uniquely identify the explanandum but cover no other instance. A unique identifier does not provide any useful new information to explain the model’s classification. For the person requiring the explanation, this outcome represents a failure of the system. The lowest failure rates (0.5%) may be tolerable, depending on the criticality or compliance requirements of the application. However, we do not foresee any circumstances where a failure rate at the upper end of this range (28%) would ever be acceptable. Secondly, such over-fitting is symptomatic of an algorithm that generates rules that are overly long; having too many terms in the antecedent to be easily interpretable. To show the link between over-fitting and rule length we present the rule length distribution in Fig 10.

We present the results of the hypothesis tests in Tables 15-16. Clearly, Anchors dominates out of the three algorithms on a statistical test of median differences. However, we have shown that these results should be taken with caution. To begin with, Anchors required us to discretise the data as a preprocessing step, which resulted in alternative models that were less accurate classifiers. The difference was two or more percentage points in 7/9 for SAMME models and 5/9 for SAMME.R models. Moreover, Anchors has a long tail distribution of rule length, and sometimes a high proportion of critically over-fitting explanations. The tabulated means of precision do not show a clear difference between Ada-WHIPS and Anchors (see Appendix). Furthermore, precision (specificity) is in a trade-off with coverage (generality). Rules that are too specific only apply to a small fraction of other instances. Ada-WHIPS makes a very small trade-off (just a percentage point or two in most cases), and delivers much more generalisable rules that rarely, if ever, over-fit. This behaviour is the result of optimising the novel stability function (Eq. 8).

**Table 15:** Precision: Top two by mean rank (mrnk) for three-way comparisons

Data	1^st^	mrnk	2^nd^	mrnk	N	*z*	*p*.value
SAMME							
Breast	**Anchors**	1.40	Ada-WHIPS	1.97	170	3.31	0.0004**
Cardiotocography	**Anchors**	1.39	Ada-WHIPS	2.09	637	7.89	≈0**
Diabetic retinography	**Anchors**	1.62	Ada-WHIPS	1.96	344	2.85	0.0022**
Cleveland heart	**Anchors**	1.16	Ada-WHIPS	2.03	90	3.68	0.0001**
Mental health survey ’16	Anchors	1.83	LORE	1.95	429	1.02	0.1539
SAMME.R							
Breast	**Anchors**	1.35	Ada-WHIPS	2.08	170	4.38	<0.0001**
Cardiotocography	**Anchors**	1.28	Ada-WHIPS	2.09	637	9.16	≈0**
Diabetic retinography	**Anchors**	1.50	Ada-WHIPS	1.92	344	3.47	0.0002**
Cleveland heart	**Anchors**	1.24	Ada-WHIPS	1.90	90	2.77	0.0028**
Mental health survey ’16	Anchors	1.83	Ada-WHIPS	1.84	429	0.08	0.4678

**Table 16:** Precision: Mean rank (mrnk) for two-way comparisons

Data	1^st^	mrnk	2^nd^	mrnk	N	*V*	*p*.value
SAMME							
Mental health survey ’14	**Anchors**	1.11	Ada-WHIPS	1.89	377	45074	≈0**
Hospital readmission	**Anchors**	1.24	Ada-WHIPS	1.76	1000	333580	≈0**
Thyroid	**Anchors**	1.19	Ada-WHIPS	1.81	1000	405600	≈0**
Understanding society	**Anchors**	1.08	Ada-WHIPS	1.92	1000	458060	≈0**
SAMME.R							
Mental health survey ’14	**Anchors**	1.11	Ada-WHIPS	1.89	377	45281	≈0**
Hospital readmission	**Anchors**	1.07	Ada-WHIPS	1.93	1000	480520	≈0**
Thyroid	Anchors	1.47	Ada-WHIPS	1.53	1000	233670	0.1601
Understanding society	**Anchors**	1.31	Ada-WHIPS	1.69	1000	266150	≈0**

### Stability analysis

**Fig. 11:**
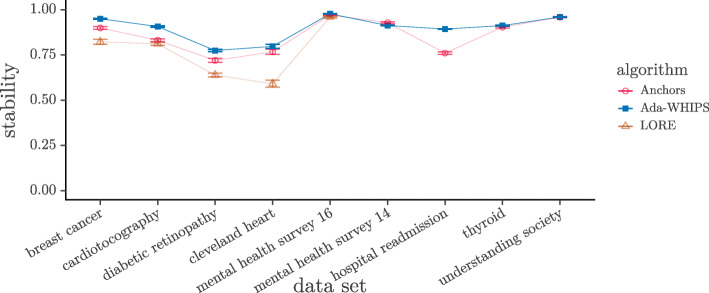
Mean Stability SAMME. Guide lines are added to mitigate over-plotting

**Fig. 12:**
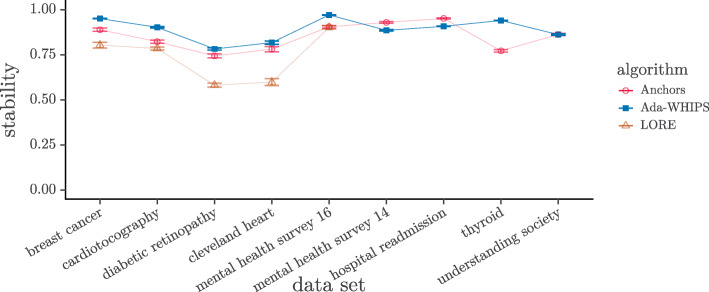
Mean Stability SAMME.R. Guide lines are added to mitigate over-plotting

Stability can also be used as a quality measure in the XAI setting. A precision of 0.0 for an explanation on a held-out test set can be caused by sampling artefacts (i.e. the ground truth may be a non-zero probability of finding certain attributes and that they are simply under-represented in the data set). For this reason, it can be argued that a precision of 0.0 is a harsh penalty against the aggregate score. Yet, if the rule covers and is correct for just a single instance in the held out set, the precision will be 1.0. This circumstance creates a discontinuity and gives a huge advantage to undesirable, over-fitting explanations. Instead of precision, we can measure stability while including the explanandum in the held out set. This condition results in the formulation $\frac {n + 1}{m + K}$ where *n* is the number of covered and correct instances, *m* is the number of covered instances and *K* is the number of classes. See Eq. (8). Thus, stability is very similar to the classical additive smoothing function (precision with Laplace correction [65]). The minimum/maximum are both $\frac {1}{1 + K}$ for *N*=1 but approach 0/1 asymptotically as *N*→*∞*. We present the visual analysis of stability in Figs. 11-12 and the results of the hypothesis tests in Tables 17-18. The post-hoc tests for the three-way comparisons show that Ada-WHIPS is the top or in the top two with no statistical difference in all except mental health survey ’16 for the SAMME model. For the two-way comparisons, Ada-WHIPS has a significantly higher rank for hospital readmission (SAMME) and thyroid (SAMME.R) but lower for the remaining results.

**Table 17:** Stability: Top two by mean rank (mrnk) for three-way comparisons

Data	1^st^	mrnk	2^nd^	mrnk	N	*z*	*p*.value
SAMME							
Breast	**Ada-WHIPS**	1.48	Anchors	2.16	170	3.96	<0.0001**
Cardiotocography	**Ada-WHIPS**	1.48	Anchors	2.19	637	7.99	≈0**
Diabetic retinography	Ada-WHIPS	1.70	Anchors	1.84	344	1.18	0.1198
Cleveland heart	Ada-WHIPS	1.60	Anchors	1.70	90	0.42	0.3374
Mental health survey ’16	Anchors	1.87	LORE	2.00	429	1.14	0.1269
SAMME.R							
Breast	**Ada-WHIPS**	1.38	Anchors	2.18	170	4.67	≈0**
Cardiotocography	**Ada-WHIPS**	1.49	LORE	2.10	637	6.80	≈0**
Diabetic retinography	Ada-WHIPS	1.64	Anchors	1.67	344	0.24	0.4050
Cleveland heart	Ada-WHIPS	1.49	Anchors	1.73	90	0.98	0.1638
Mental health survey ’16	**Ada-WHIPS**	1.44	LORE	2.18	429	6.45	≈0**

**Table 18:** Stability: Mean rank (mrnk) for two-way comparisons

Data	1^st^	mrnk	2^nd^	mrnk	N	*V*	*p*.value
SAMME							
Mental health survey ’14	**Anchors**	1.19	Ada-WHIPS	1.81	377	39293	≈0**
Hospital readmission	**Ada-WHIPS**	1.43	Anchors	1.57	1000	136050	≈0**
Thyroid	**Anchors**	1.35	Ada-WHIPS	1.65	1000	307840	≈0**
Understanding society	**Anchors**	1.14	Ada-WHIPS	1.86	1000	405340	≈0**
SAMME.R							
Mental health survey ’14	**Anchors**	1.19	Ada-WHIPS	1.81	377	40515	≈0**
Hospital readmission	**Anchors**	1.14	Ada-WHIPS	1.86	1000	439750	≈0**
Thyroid	**Ada-WHIPS**	1.18	Anchors	1.82	1000	50600	≈0**
Understanding society	**Anchors**	1.39	Ada-WHIPS	1.61	1000	220150	≈0**

### Efficiency analysis

**Fig. 13:**
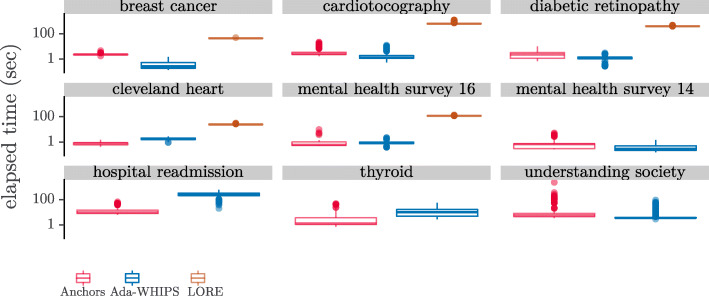
Distributions of Computation Time per Explanation. Note the y-axis is log10 scaled

Finally, we show the distribution of computation time per explanation in Fig. 13. A brief visual inspection shows that Ada-WHIPS and Anchors are roughly comparable for all data sets. The shortest run-times are fractions of a second and the longest are two to three minutes. LORE runs at several orders of magnitude longer than this. As we discussed in previous sections, it was prohibitive to run LORE for the data sets mental health survey ’14, hospital readmission, thyroid and understanding society with a single explanation taking over two hours to generate. We performed both static and dynamic analysis of the LORE source code and discovered that the bottleneck was in a non-parallelisable, genetic-algorithmic step.

## Discussion

### Advantages of Ada-WHIPS

Our method improves on prior research in that it delivers explanations that have high mean coverage (15%-68%). Ada-WHIPS explanations generalise well while making only a very small trade-off to keep precision/specificity competitive (80%-99%). At the same time, Ada-WHIPS is guarded against over-fitting while competing methods have the tendency to present critically over-fitting explanations, in 0.05%-28% of cases. A critically over-fitting explanation is defined as an explanation that uniquely identifies the explanandum and covers no other instances. Ada-WHIPS does not make any assumptions about the underlying data distribution, while some competing methods require continuous features to be discretised prior to model training. This treatment of the data can result in a less accurate model, detracting from the main benefit of using AdaBoost at the outset. By design, Ada-WHIPS rules extract discrete, logical conditions from the base decision tree classifiers of the AdaBoost model. These logical conditions have an information-theoretic derivation and we speculate that this is what leads to Ada-WHIPS’s favourable trade-off between precision and coverage. Ada-WHIPS is efficient. At its fastest, explanations are generated in fractions of seconds. On high dimensional data sets, we recorded times of up to three minutes per explanation. This is in line with competing methods and could still be considered real-time in the context of a medical consultation. As a minor contribution, we presented stability, a novel measure that is a regularised version of precision. It gives more informative results in the XAI setting as it penalises low coverage while correcting for sampling artefacts.

### Limitations of Ada-WHIPS

By design, Ada-WHIPS is a companion method for AdaBoost models and the algorithm is not transferable to other models without adaptation. In contrast, model-agnostic methods, such as Anchors and LORE, can be applied to any black box model with few restrictions. It is up to the end user to determine which approach best suits their specific scenario. Ada-WHIPS is an heuristic method for finding a short rule with high coverage and precision. Consequently, Ada-WHIPS will not provide a feature attribution value for each attribute with theoretical guarantees. If such values with guarantees are required, then the combinatorial calculation of Shapley Values is the recommended method.

### Challenges

Experimental studies of XAI are challenging in terms of their time cost. Each explanation must be generated individually and, for all currently well-cited methods, generation of explanations is a much more time consuming process than the classification step. Furthermore, each explanation must be evaluated individually, rather than batchwise. For example, a trivial confusion matrix or AUC-ROC test is not appropriate. We calculated scores for each explanation and then used the means, medians and mean ranks to compare methods. Any experimental design for evaluating XAI must allow for this time cost, and also consider how instances used to generate explanations can be separated from instances used to evaluate explanations. Such designs may require three data partitions (training, explanation generating, explanation evaluating). We opted for a leave-one-out procedure, training a model on a training set then generating explanations one at a time and evaluating on the remaining instances from a held-out set.

## Conclusion & future work

Our main contribution is the novel algorithm Ada-WHIPS for explaining the classification of AdaBoost models with simple classification rules. AdaBoost models are widely adopted as computer aided diagnostic tools and the non-clinical identification of sub-health and mental health conditions using unconventional data sources such as online health communities. As a minor contribution, we propose stability as a novel function for optimisation of explanation algorithms that explicitly avoids over-fitting and can be used as a quality metric in evaluations of XAI experimental research.

Directions for future work include developing the method for Gradient Boosting Machines such as XGBoost that use decision trees as the base classifiers, and applying the proposed method on a variety of healthcare and medical data sets.

## Appendix

## Supplementary

### Cohen’s *κ*

Cohen’s *κ* is calculated as:

10 $$ {{}\begin{aligned} \kappa = \frac{N \sum^{K}_{i=1} N_{ii} - \sum^{K}_{i=1} N_{i+} N_{+i} }{N^{2} - \sum^{K}_{i=1} N_{i+} N_{+i}},\ \left[\begin{array}{cccc} \mathrm{N}_{11} & \mathrm{N}_{12} & \dots & \mathrm{N}_{1K} \\ \mathrm{N}_{21} & \mathrm{N}_{22} & \dots & \mathrm{N}_{2K} \\ \vdots & \vdots & \ddots & \vdots \\ \mathrm{N}_{K1} & \mathrm{N}_{K2} & \dots & \mathrm{N}_{KK} \end{array}\right] \end{aligned}}  $$

where *K* is the number of classes, *N* is the total number of instances, *N*_*ij*_ is the number of instances in cell *ij* of the confusion matrix of true vs. predicted class counts, and *N*_*i*+_,*N*_+*j*_ are the *i*^*th*^ row and *j*^*th*^ column marginal totals, respectively.

**Table 19:** Coverage of explanations of AdaBoost SAMME

Data	Ada-WHIPS	Anchors	LORE
Breast cancer	0.3635±0.0068	0.1530±0.0053	0.3914±0.0156
Cardiotocography	0.3867±0.0092	0.0637±0.0018	0.4417±0.0120
Diabetic retinopathy	0.3225±0.0125	0.0636±0.0039	0.1060±0.0060
Cleveland heart	0.2310±0.0084	0.1101±0.0079	0.3259±0.0259
Mental health survey ’16	0.4974±0.0026	0.3915±0.0083	0.3777±0.0086
Mental health survey ’14	0.3368±0.0063	0.1483±0.0030	N/A
Hospital readmission	0.1809±0.0040	0.0095±0.0004	N/A
Thyroid	0.3630±0.0074	0.0636±0.0015	N/A
Understanding society	0.6679±0.0108	0.2729±0.0040	N/A

### Friedman test

The original Friedman test produces an approximately *χ*^2^ distributed statistic, but this is known to be very conservative. Therefore, we use the modified F-test given in [64], because we have very large values for *N*, i.e. the count of instances in the test set. The null hypothesis of this test is that there is no significant difference between the mean ranks *R* of all the groups and the alternative is that at least two mean ranks are different. The null hypothesis is rejected when *F*_*F*_ exceeds the critical value for an *F* distributed random variable with the first degrees of freedom *df*_1_=*k*−1 and the second *df*_2_=(*k*−1)(*N*−1), where *k* is the number of algorithms:

11 $$ \begin{aligned} F_{F} = \frac{(N - 1) \chi^{2}_{F}}{N(k-1) - \chi^{2}_{F}},\ \ \chi^{2}_{F} = \frac{12N}{k(k+1)}\left[ \sum^{k}_{j=1}{R_{j}^{2} - \frac{k(k+1)^{2}}{4}}\right] \end{aligned}  $$

**Table 20:** Coverage of explanations of AdaBoost SAMME.R

Data	Ada-WHIPS	Anchors	LORE
Breast cancer	0.33502±0.0055	0.1513±0.0054	0.3574±0.0157
Cardiotocography	0.3894±0.0093	0.0667±0.0019	0.4765±0.0128
Diabetic retinopathy	0.1349±0.0053	0.0759±0.0040	0.0945±0.0068
Cleveland heart	0.2182±0.0085	0.1180±0.0078	0.3754±0.0271
Mental health survey ’16	0.3578±0.0054	0.1778±0.0072	0.3248±0.0101
Mental health survey ’14	0.2927±0.0053	0.1444±0.0030	N/A
Hospital readmission	0.1598±0.0038	0.1345±0.0042	N/A
Thyroid	0.3793±0.0073	0.0224±0.0008	N/A
Understanding society	0.6891±0.0107	0.1057±0.0038	N/A

The recommended pairwise, post-hoc comparison test with the Bonferroni correction (for three pairwise comparisons) proposed in [64]:

12 $$ z = \text{diff}_{ij} \bigg/ \sqrt{\frac{k(k+1)}{6N}},\ \text{diff}_{ij} = R_{i} - R_{j}  $$

**Table 21:** Precision of explanations of AdaBoost SAMME

Data	Ada-WHIPS	Anchors	LORE
Breast cancer	0.9819±0.0022	0.9915±0.0062	0.8405±0.0179
Cardiotocography	0.9369±0.0039	0.9915±0.0097	0.8209±0.0109
Diabetic retinopathy	0.8031±0.0075	0.8016±0.0188	0.6300±0.0182
Cleveland heart	0.8744±0.0118	0.9644±0.0189	0.6300±0.0321
Mental health survey ’16	0.9862±0.0010	0.9873±0.0035	0.9744±0.0061
Mental health survey ’14	0.9301±0.0021	0.9798±0.0056	N/A
Hospital readmission	0.8973±0.0016	0.8163±0.0110	N/A
Thyroid	0.9205±0.0026	0.9441±0.0055	N/A
Understanding society	0.9643±0.0016	0.9749±0.0035	N/A

**Table 22:** Precision of explanations of AdaBoost SAMME.R

Data	Ada-WHIPS	Anchors	LORE
Breast cancer	0.9831±0.0014	0.9793±0.0103	0.8215±0.0210
Cardiotocography	0.9324±0.0032	0.9117±0.0107	0.7931±0.0110
Diabetic retinopathy	0.8272±0.0073	0.8164±0.0175	0.5481±0.0203
Cleveland heart	0.9059±0.0105	0.9640±0.0189	0.5971±0.0293
Mental health survey ’16	0.9849±0.0013	0.9502±0.0100	0.9129±0.0124
Mental health survey ’14	0.9030±0.0043	0.9811±0.0056	N/A
Hospital readmission	0.9129±0.0013	0.9811±0.0032	N/A
Thyroid	0.9481±0.0015	0.8154±0.0110	N/A
Understanding society	0.8677±0.0043	0.8903±0.0081	N/A

where *R*_*i*_ and *R*_*j*_ are ranks of two algorithms and *z* is distributed as a standard normal under the null hypothesis that the pair of ranks are not significantly different. The critical value for a two-tailed test with the bonferroni correction is $\frac {0.025}{3} = 0.00833$

**Table 23:** Stability of explanations of AdaBoost SAMME

Data	Ada-WHIPS	Anchors	LORE
Breast cancer	0.9500±0.0024	0.8992±0.0072	0.8226±0.0137
Cardiotocography	0.9067±0.0044	0.8311±0.0078	0.8113±0.0085
Diabetic retinopathy	0.7745±0.0067	0.7196±0.0114	0.6388±0.0106
Cleveland heart	0.7973±0.0106	0.7671±0.0145	0.5906±0.0195
Mental health survey ’16	0.9770±0.0011	0.9706±0.0053	0.9592±0.0046
Mental health survey ’14	0.9125±0.0021	0.9283±0.0053	N/A
Hospital readmission	0.8930±0.0017	0.7306±0.0071	N/A
Thyroid	0.9121±0.0028	0.9033±0.0047	N/A
Understanding society	0.9594±0.0017	0.9586±0.0035	N/A

**Table 24:** Stability of explanations of AdaBoost SAMME.R

Data	Ada-WHIPS	Anchors	LORE
Breast cancer	0.9505±0.0017	0.8885±0.0089	0.8035±0.161
Cardiotocography	0.9020±0.0038	0.8226±0.0087	0.7844±0.0086
Diabetic retinopathy	0.7821±0.0064	0.7436±0.0109	0.5814±0.0111
Cleveland heart	0.8171±0.0092	0.7807±0.0143	0.5985±0.0190
Mental health survey ’16	0.9707±0.0015	0.9051±0.0073	0.9013±0.0088
Mental health survey ’14	0.8852±0.0041	0.9293±0.0051	N/A
Hospital readmission	0.9075±0.0029	0.9514±0.0029	N/A
Thyroid	0.9401±0.0015	0.7716±0.0071	N/A
Understanding society	0.8616±0.0043	0.8624±0.0063	N/A

## Data Availability

The source code and data sets analysed during the current study are available in our repository: https://tinyurl.com/yxuhfh4e.

## Abbreviations

Ada-WHIPS: Adaptive-weighted high importance path snippets
AFAM: Additive feature attribution methods
BRL: Bayesian rule list
CAD: Computer aided diagnostics
CBR: Case-based reasoning
CR: Classification rule
CRL: Cascading rule list
DT: Decision tree(s)
EHR: Electronic health record(s)
GAM: Generalised additive model(s)
GA^2^M: Generalised additive model(s) with second order interactions
KL: Kullback-Leibler (divergence)
LIME: Local interpretable model-agnostic explanations
LORE: LOcal rule-based explanations
MDS: Multi-dimensional scaling
ML: Machine learning
PI: Partial independence (plots)
RNN: Recurrent neural network
SAMME: Stagewise additive modeling using a multi-class exponential loss function
SAMME.R: Real-valued SAMME
SHAP: SHapley additive exPlanations
SVM: Support vector machine(s)
TSH: Thyroid stimulating hormone
WkNN: Weighted k-nearest neighbours
XAI: eXplainable artificial intelligence

## Footnotes

1 https://tinyurl.com/qlyxzlv
